# High-Throughput Pseudovirion-Based Neutralization Assay for Analysis of Natural and Vaccine-Induced Antibodies against Human Papillomaviruses

**DOI:** 10.1371/journal.pone.0075677

**Published:** 2013-10-04

**Authors:** Peter Sehr, Ivonne Rubio, Hanna Seitz, Kerstin Putzker, Lis Ribeiro-Müller, Michael Pawlita, Martin Müller

**Affiliations:** 1 EMBL-DKFZ Chemical Biology Core Facility, European Molecular Biology Laboratory, Heidelberg, Germany; 2 Infections and Cancer Program, German Cancer Research Center (DKFZ), Heidelberg, Germany; Centers for Disease Control and Prevention, United States of America

## Abstract

A highly sensitive, automated, purely add-on, high-throughput pseudovirion-based neutralization assay (HT-PBNA) with excellent repeatability and run-to-run reproducibility was developed for human papillomavirus types (HPV) 16, 18, 31, 45, 52, 58 and bovine papillomavirus type 1. Preparation of 384 well assay plates with serially diluted sera and the actual cell-based assay are separated in time, therefore batches of up to one hundred assay plates can be processed sequentially. A mean coefficient of variation (CV) of 13% was obtained for anti-HPV 16 and HPV 18 titers for a standard serum tested in a total of 58 repeats on individual plates in seven independent runs. Natural antibody response was analyzed in 35 sera from patients with HPV 16 DNA positive cervical intraepithelial neoplasia grade 2+ lesions. The new HT-PBNA is based on Gaussia luciferase with increased sensitivity compared to the previously described manual PBNA (manPBNA) based on secreted alkaline phosphatase as reporter. Titers obtained with HT-PBNA were generally higher than titers obtained with the manPBNA. A good linear correlation (R^2^ = 0.7) was found between HT-PBNA titers and anti-HPV 16 L1 antibody-levels determined by a Luminex bead-based GST-capture assay for these 35 sera and a Kappa-value of 0.72, with only 3 discordant sera in the low titer range. In addition to natural low titer antibody responses the high sensitivity of the HT-PBNA also allows detection of cross-neutralizing antibodies induced by commercial HPV L1-vaccines and experimental L2-vaccines. When analyzing the WHO international standards for HPV 16 and 18 we determined an analytical sensitivity of 0.864 and 1.105 mIU, respectively.

## Introduction

Human papillomaviruses (HPV) are causally involved in the induction of cervical cancer and its precursor lesions. Currently, 12 HPV types are classified as carcinogenic to humans and an additional 8 types as probably or possibly carcinogenic to human [1]. Worldwide, the ten HPV types identified most frequently in cervical cancer are HPV 16, 18, 33, 45, 31, 58, 52, 35, 59 and 56 [2]. HPV infection is recognized as an absolute requirement for the transformation process in cervical cancer [3], [4], but host cell cofactors also play a role. Built on the recognition of the HPV causality in cervical cancer development, two commercial vaccines, Gardasil® and Cervarix® targeting the two most prevalent carcinogenic HPV types 16 and 18 were licensed in the EU in 2006 and 2007, respectively [5], [6]. Both vaccines employ the major capsid protein L1 in form of virus-like particles (VLPs) as antigen and are highly effective in preventing infections by HPV types 16 and 18 as well as cervical intraepithelial neoplasias induced by these viruses [7], [8].

The mode of action of both vaccines is considered to be the induction of neutralizing antibodies directed against L1 surface loops of the viral capsid. With more than six years history on papillomavirus prophylactic vaccination, monitoring long term development of protective titers of neutralizing antibodies is of increasing importance. Thus, there is a need for the evaluation of such antibody responses, specifically for functional assays analyzing neutralizing antibodies.

Papillomaviruses cannot be replicated in simple cell culture systems. Therefore, in the past a number of functional assays have been developed to measure antibody-mediated neutralization of papillomaviruses. These assays involved the use of authentic viruses [9] [10] and so called pseudovirions with an encapsidated reporter construct [11], [12], [13]. In addition, neutralizing antibodies have been measured more indirectly e.g. by a hemagglutination inhibition assay [14] or by competition of binding of a neutralizing monoclonal antibody [15].

The current ‘gold standard’ for measuring neutralizing anti-HPV antibodies is a manually performed pseudovirion-based neutralization assay (manPBNA; [16]) using secreted alkaline phosphatase (SEAP) as reporter. Although infectious pseudovirions of different PV types can be easily produced, the manPBNA remains tedious and variable, restricting its applicability mainly to small sample numbers.

Several arguments make a case for the need of a high-throughput neutralization assay with improved sensitivity: (i) requirement of larger serum sample numbers for follow-up studies on current vaccines, (ii) detection of cross-neutralizing antibodies induced by the commercial vaccines, and (iii) monitoring the effect of simplified vaccination schemes. Also, induction of neutralizing antibodies by second generation vaccines, e.g. based on the L2 protein needs to be assessed. Finally, large scale neutralization assays would allow addressing questions on naturally occurring protective immunity against HPV infections. Especially in respect to antibody responses against natural papillomavirus infections there are high demands for sensitivity and reproducibility in a neutralization assay. To date, high-throughput detection of HPV capsid-specific antibodies has been possible only with the aid of surrogate detection assays such as ELISA and competitive Luminex immunoassay (cLIA) using VLPs as antigen. Here we describe the adaptation of the PBNA to a high-throughput (HT) setting. We developed a purely add-on system in which the serial dilution of serum samples is separated from the cell-based assay, providing a high degree of flexibility. The high-throughput assay demonstrates high robustness with little intra- and inter-assay day variability. Also, the HT-PBNA shows higher sensitivity compared to the manually performed assay using SEAP as reporter. In its current format, the neutralization titer of 110 serum samples for seven HPV types can be determined in a single run. The HT-PBNA will allow the execution of large studies on vaccine- and naturally-induced immunity against HPV infections.

## Materials and Methods

### Human Sera

Base line sera from 35 women with HPV 16 mono-infected high grade cervical intraepithelial neoplasia (CIN 2/3) participating in a vaccination trial with chimeric HPV 16 L1-E7 virus-like particles [17] were used as examples of naturally acquired HPV 16 antibodies. For vaccine-induced HPV 16 antibodies, 72 post-vaccination sera of this vaccination trial were used. **Ethics statement** [17]: From December 2000 until May 2002, 39 patients were enrolled into the study at 10 German study sites (Berlin, Duesseldorf, Hamburg, Hanover, Jena, Mainz, Munich (2), Muenster, Rostock). Positive votes were obtained from each internal review board of the participating centers. Ethics regulations were coordinated by the ‘Ethik-Kommission der Friedrich-Schiller-Universität Jena an der Medizinischen Fakultät’ (http://www.ethikkommission.uniklinikum-jena.de/Ethik_KommissionderFSUJena.html). All investigations involving humans have been performed in accordance with the principles embodied in the Declaration of Helsinki. The study was performed in compliance with the guidelines of Good Clinical Practice as described in the International Conference on Harmonization of Good Clinical Practices guidelines step 4 (1996). All patients signed an informed consent form. Study participation took 24 weeks with 2 optional follow-up visits after 36 and 48 weeks. Blood samples were collected at baseline, 2 weeks after each vaccination and after 24 weeks. Peripheral blood mononuclear cells (PBL) and plasma were stored frozen until analysis.

A post-vaccination serum collected after obtaining written informed consent from a 21 years old female volunteer 3 years post completion of a 3-dose Gardasil vaccination course was used as positive standard on all assay plates. Approval for obtaining and testing this serum derives from ‘Ethik-Kommission der Bayerischen Landesärztekammer’, Munich, Germany.

### HPV Pseudovirion Preparation

Pseudovirions (PSV) were prepared as previously described [18], [19] with some modifications. Pseudovirions were prepared by co-transfecting 293TT cells with plasmids encoding humanized HPV L1 and L2 genes. For HPV 16 we used a bi-cistronic L1 and L2 expression construct, exceeding packagable size. For Production of HPV 18 and BPV 1 PSV we used two plasmids encoding L1 and L2 separately. As a reporter we used a plasmid encoding the enzyme Gaussia luciferase or a plasmid encoding secreted human placental alkaline phosphatase (SEAP). Co-transfection was performed on 4×10^6^ 293TT cells using TurboFect (Fermentas) according to the manufacturer’s instructions with minor modifications. Briefly, 10–15 µg DNA were mixed with 2 volumes of TurboFect and 1 ml of unsupplemented DMEM and added to 293TT cells which were subsequently incubated at 37°C, 5%CO_2_ for 72 hours. Absence of cell culture contaminations and authentication of the cell line were determined by the Multiplex cell Contamination Test [20] and a SNP-based human cell line authentication test as described [21].

For pseudovirion extraction, 293TT cells were harvested and the cell pellet was resuspended in an equal volume of lysis buffer. Lysis buffer was prepared by adding 58,3 µl of 10% Brij58 (w/v) (Sigma) and 6,6 µl RNAse A/T (Fermentas) to every ml of DBPS (Invitrogen). Cells were rotated overnight at 37°C to induce pseudovirion maturation. On the next day, salt extraction was performed by adding 0,17 volumes of 5 M NaCl, followed by the addition of 250 units of benzonase for 1 h at 37°C. The pseudovirions were purified by Optiprep gradient and fractions were stored at −80°C.

### manPBNA

The SEAP manPBNA was essentially performed as described [16]. As target cells 293TT cells were seeded at a concentration of 15,000 cells per well on 96-well plates in DMEM (Sigma), supplemented with 10% FCS (Gibco, BRL), 1% penicillin/streptomycin (Life Technologies) and 125 µg/ml hygromycin (Roche). The following day, the pseudovirions were diluted in DMEM (1∶5000) and mixed with the sera at different dilutions. After 15 min incubation at room temperature, the medium of the 293TT cells was replaced by 200 µl of the pseudovirion solution. The following controls were included: untreated cells, cells treated with pseudovirions alone, and cells treated with pseudovirions in the presence of neutralizing monoclonal antibodies. Detection of SEAP in the cell culture supernatant was performed five days later with the chemiluminescent SEAP Reporter Gene Assay (Roche) according to the manufacturer’s instructions. All sera were tested in duplicates. The neutralization activity was calculated using the following formula: % neutralization = (Psv_alone_−Psv_serum_)/(Psv_alone_−Psv_Ab_)*100%. Sera with at least 70% neutralizing activity were regarded as neutralizing.

### Luminex GST-L1 Capture Serology

Antibodies binding to HPV L1 proteins were measured in a multiplexed glutathione S-transferase capture immunosorbent assay with fluorescent bead-based Luminex technology as previously described in detail [22], [23] [24], [25]. The cut off for the Luminex assay was calculated from the reactivity of pre-vaccine sera that were negative at 1∶100 dilution (<109 MFI). At the 1∶2700 dilution that was used to measure MFI-values in a linear range for the vaccine sera, the maximum reactivity of the above mentioned negative pre-vaccine sera was 10 MFI and therefore used as cut off value for the sera at 1∶2700 dilution.

### Reporter Cell Line HeLaT for HT-PBNA

The 293TT cells originally described as target cell line for the PBNA were not suited for the add-on assay layout for the HT-PBNA since attachment of the 293TT cells to the assay plates was very variable and resulted in high assay variability. Therefore, we generated a HeLa cell line stably transfected with a linearized expression construct for the SV 40 large T-antigen. HeLa cells (tested for absence of contaminations and for authenticity as described above [20]) were transfected and then cultivated under selection using hygromycin B. Several single clones were isolated and tested for transduction by HPV 16 pseudovirions. One clone (HeLaT clone-4) was subsequently used in all assays.

### High-throughput Pseudovirion-based Neutralization Assay (HT-PBNA)

#### A. Assay plate preparation

**Figure 1:**
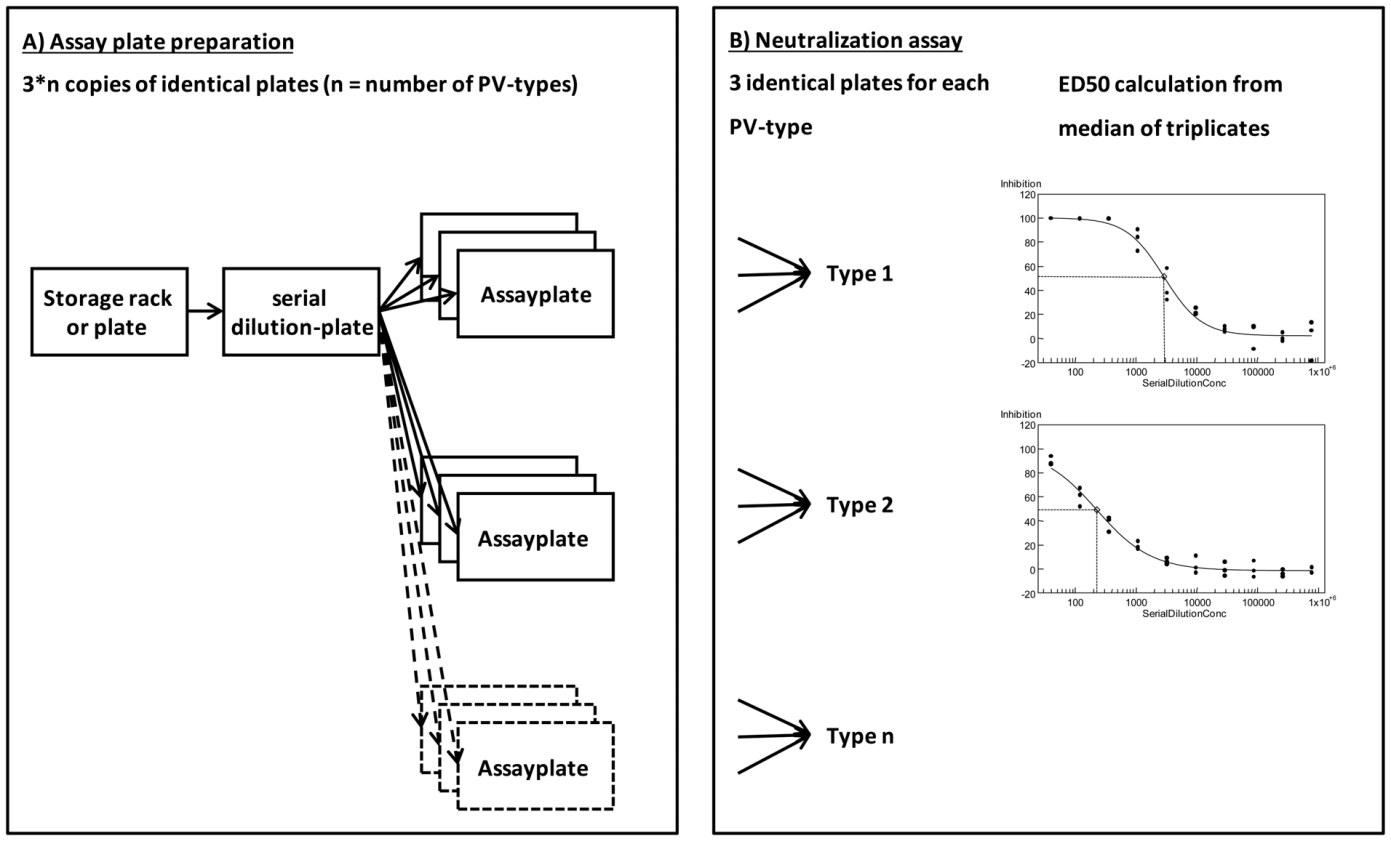
HT-PBNA protocol. Assay plate preparation (A) and neutralization assay (B) are separated. In a first step, serial dilutions of serum samples are performed on one dilution plate. Identical assay plates are generated by transferring the dilutions to multiple replica-assay plates which can be stored at −20°C. In the second step, the neutralization assay is carried out in a add-on format using the previously prepared assay plates. **A) Assay plate preparation**. Sera are transferred from a 96 well storage in SBS standard to a 384 well polypropylene V-bottom plate for serial dilution with a pipetting robot in cell culture medium. Finally the serially diluted sera are transferred with the same pipetting robot to each of 3*n white 384 well cell culture assay plates (n = number of PV-types). The plates are sealed immediately with a cover foil and stored at −20°C until their use in the PBNA. **B) Assay assembly and read out**. Assay plates are thawed and pseudovirions followed by reporter cells are added with a bulk dispenser. Three identical assay plates originating from the same serum dilution plate are used for each PV- type. After 2 days of incubation the luminescence from the Gaussia reporter is read directly in the assay plates. Inhibition (%) is calculated by normalization of the luminescence to the mean of the negative control wells without serum present on each plate. The median of the triplicate values is used for the calculation of the ED_50_-value (effective dilution giving 50% inhibition) for each serum according to the four parameter logistic curve fit model y = A+(B−A)/(1+(C/x)^∧^D).

In the automated high-throughput set up all sample racks and plates were barcoded to ensure continuous sample and data tracking with the help of pipetting robots and plate readers equipped with barcode readers. Sera were transferred from a 96 well storage in SBS standard format (http://www.slas.org/community/microplate_standards.cfm) to columns 3 and 13 of 384 well intermediate plates (polypropylene V-bottom plates, Greiner; figure 1) using a Perkin Elmer MPII 4-tip automated liquid handling robot and then 10-fold serially diluted with an EP3 robot (384 channel head) in DMEM cell culture medium (Invitrogen) supplemented with 10% fetal calf serum (Gibco) and Penicillin/Streptomycin (Life Technologies). Starting from the initial dilution in column 3 the sera were transferred from column n to column n+1 containing cell culture medium and the solution mixed after each transfer on a Variomag Teleshake (Inheco, Germany) for 45 sec at 1300 rpm.

The initial dilution, transferred and provided volumes depend on the desired starting concentration, the step width of the serial dilution and the number of replicates that have to be prepared from the serial dilution. Sera from the vaccination trial with chimeric HPV 16 L1-E7 virus-like particles were diluted in 3-fold increments starting from 1/5 (18 µl serum +72 µl medium) to 1/157,464 and finally 5 µl of the serial dilutions were transferred with the EP3 robot to each of nine assay plates (white 384 well cell culture plates, PerkinElmer). The plates were sealed immediately with a polypropylene cover foil (HJ-Bioanalytik, Germany) and stored at −20°C.

#### B. Assay assembly and read out

Assay plates containing the serum dilutions were equilibrated to room temperature (RT), centrifuged for 1 min at 1.000×g and the cover foil was removed before 15 µl PSV diluted in fully supplemented cell culture medium were added with a FlexDrop bulk dispenser (PerkinElmer) and incubated for 1 h at RT (Figure 1). Thereafter 20 µl HeLaT cells were added with the FlexDrop dispenser to give a density of 1500 cells/well and incubated for 2 days in a cell incubator at 37°C/5% CO_2_. The plates where then removed from the incubator and equilibrated to RT before 20 µl/well glow substrate solution (PJK, Germany) for the Gaussia luciferase was added with the FlexDrop dispenser. Luminescence was read directly in the assay plates after 11 min in an Envision plate reader (PerkinElmer) equipped with stacker and internal barcode reader.

Three identical assay plates originating from the same serum dilution plate were used for each PSV- type. Inhibition (%) was calculated by normalization of the luminescence in serum-dilution containing wells to the mean of the negative control wells without serum present on each plate. The median of the triplicate values was used for the calculation of the ED_50_-value (effective dilution giving 50% inhibition) for each serum according to the four parameter logistic curve fit model: y = A+(B−A)/(1+(C/x)^∧^D) (model 205 from the functions available in XE-Designer, IDBS, United Kingdom).

Plasmids and cell lines are available on request for non-commercial users following standard material transfer agreement.

## Results

In order to translate the (manual) PBNA to high-throughput format we first identified, then optimized and standardized critical parameters of the assay including pseudovirion (PSV) concentration, serum-PSV incubation time, cell lines and cell density.

### Effect of Cell Type and Density on Reporter Signal

**Figure 2:**
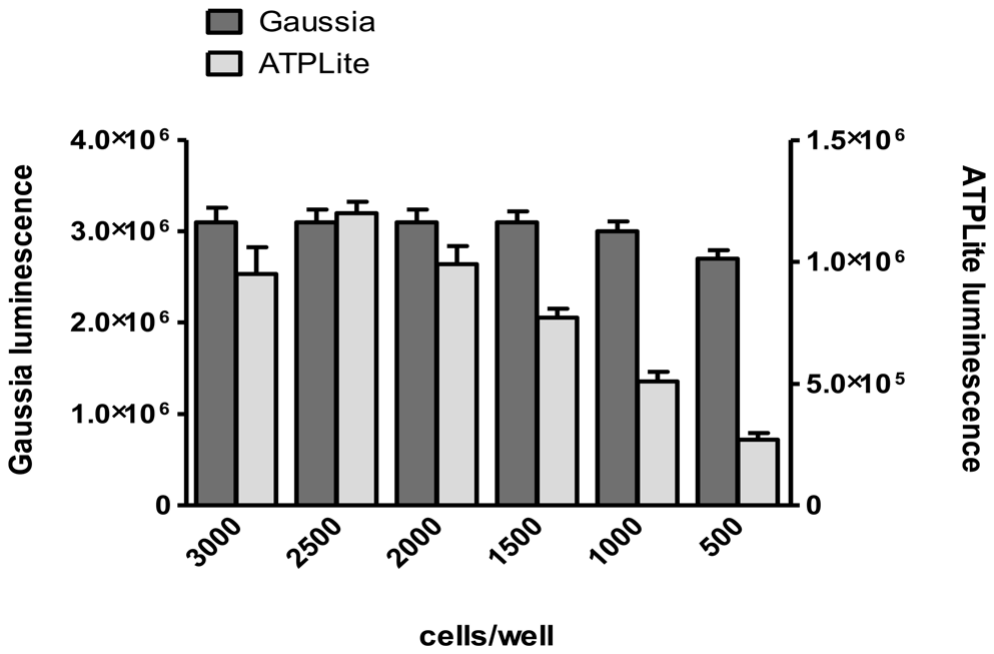
Influence of cell density on Gaussia luciferase activity. HeLaT clone 4 cells were seeded at the indicated densities into white 384 well plates containing HPV 16°C for 2 days substrate for the Gaussia luciferase or ATPLite1step™ was added and the luminescence measured. Columns show the median luminescence of 64 wells with bars indicating the standard deviation.

In preceding experiments we tested different cell lines in the HT-PBNA. The 293TT cell line [13] appeared ill-suited for the add-on assay layout of the HT-PBNA, where trypsin-treated cells are added to premixed PSV and sera. 293TT cell attachment to the assay plates was highly variable and relatively slow (>4 hours for >90% cell attachment) causing a significant delay in reporter gene expression. In contrast, more than 90% of cells of a newly established HeLa subline (HeLaT clone-4) stably expressing the SV40 large T-antigen were found to attach within 1 hour of seeding (data not shown). With the HT-PBNA protocol, a range of 500–3000 HeLaT clone-4 cells per well in the 384-well assay plates showed little influence on the intensity of the Gaussia luciferase signal (Figure 2). The luminescence signal in an ATPlite1step™ assay (Perkin Elmer) is directly correlated with cell number/metabolic activity and was proportional to the number of seeded cells, up to 2500 cells/well when confluency was reached after 2 days of incubation. In all subsequent assays, 1500 cells/well were used to ensure that cell density is not exceeding sub-confluency. The robustness of the PBNA towards variations in cell number is crucial for the application in a 384 well format where “edge effects” of the outer most wells often influence cell growth.

Typically, for the luminescence signal of the Gaussia luciferase reporter assay in 384 well plates we observed a coefficient of variation (CV) of less than 3% within rows and less than 6% for the whole plate (data not shown). The higher CV for the whole plate is mainly a consequence of the decay of the Gaussia luciferase luminescence during the time that is necessary to read the 384 well plate (75 sec), even when employing a glow substrate with reduced turnover rates. The luminescence reader measures the plate row-wise so that the decrease in signal is negligible within a row. Sera are diluted within a row and therefore the effect of a decreasing luminescence signal on the calculation of the ED_50_ is minimal and no correction for position effects is necessary.

### Pseudovirion Concentration

**Figure 3:**
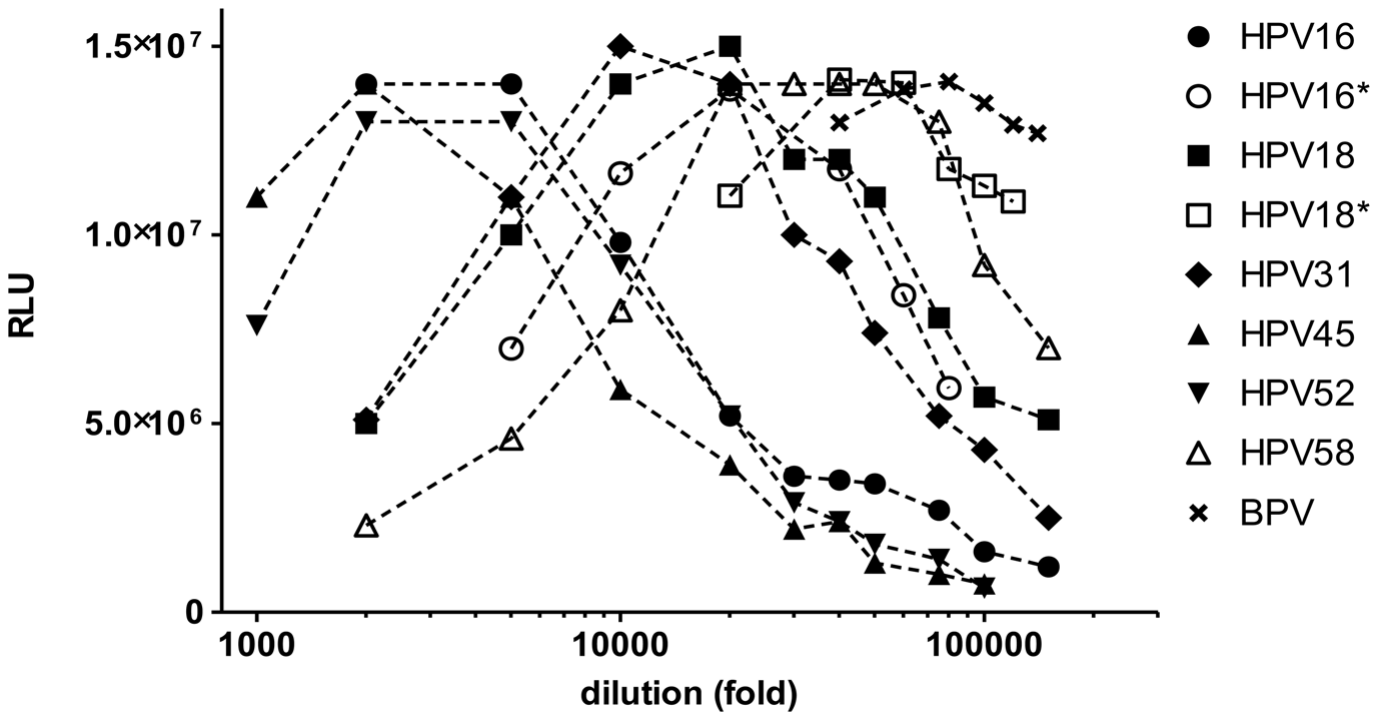
Titration of different PSV preparations. Serial dilution of PSV preparations from HPV types 16, 18, 31, 45, 52, 58 and BPV-1 were assayed for Gaussia luciferase activity in the HT-PBNA. For HPV types 16 and 18 a second PSV preparation is indicated by an asterisk (*). Luminescence signals are expressed as relative light units (RLU). The titers for undiluted HPV 16, 18, and BPV-1 PSV were 3.0×10^9^, 3.9×10^9^, 1.1×10^10^ genomes per ml, respectively.

**Figure 4:**
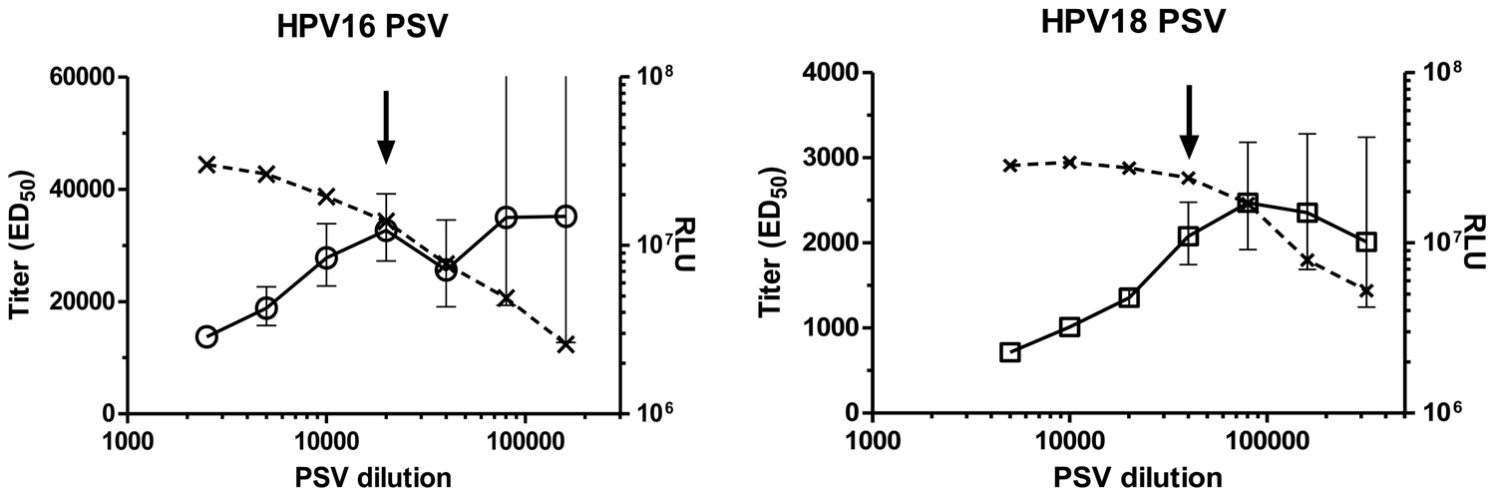
Influence of PSV concentration on HT-PBNA analytical sensitivity and robustness. Neutralization titers of a serum from a Gardasil® immunized individual expressed as ED_50_ values (open circles and open squares) with the variability (bars indicating the 95% confidence intervals) were determined at different PSV concentrations. The maximal luminescence intensities (RLU) obtained without serum are shown as crosses. An arrow indicates the dilution of the PSV preparation that was used in subsequent neutralization assays.

PSV preparations of the same or different papillomavirus types can vary considerably in regard to particle concentration, L1 capsid protein to reporter plasmid ratio and transduction activity. A standardization of PSV preparations by L1 or plasmid concentration is therefore not the best indicator of assay performance. Titration of PSV preparations in the HT-PBNA demonstrated significant variation (Figure 3). At high PSV concentrations signals were frequently reduced. All PSV preparations reached similar maximal intensities but the dilutions giving maximal luminescence signal spanned a wide range even for PSV preparations of the same HPV type (e.g. HPV 16 PSV in Figure 3). To investigate the effect of PSV dilution on sensitivity and variability of the PBNA we determined the effective PSV dilution yielding 50% neutralization (ED_50_) of a serum derived from a Gardasil-immunized individual for different dilutions of HPV 16 and HPV 18 PSV (Figure 4). As expected, lowering the PSV concentrations raised the ED_50_ values indicating an increasing analytical sensitivity. At low PSV concentrations the ED_50_ reached a plateau. The maximal gain in sensitivity was typically 3-fold relative to the highest PSV concentrations. However, the variability of the ED_50_ determination markedly increased with lower PSV concentrations, indicating reduced assay robustness. Therefore, PSV concentrations slightly lower than those generating maximal luminescence intensity were considered to represent the best compromise between low variability and high analytical sensitivity and were used in all future HT-PBNA.

### Pre-incubation Time of PSV and Sera

**Figure 5:**
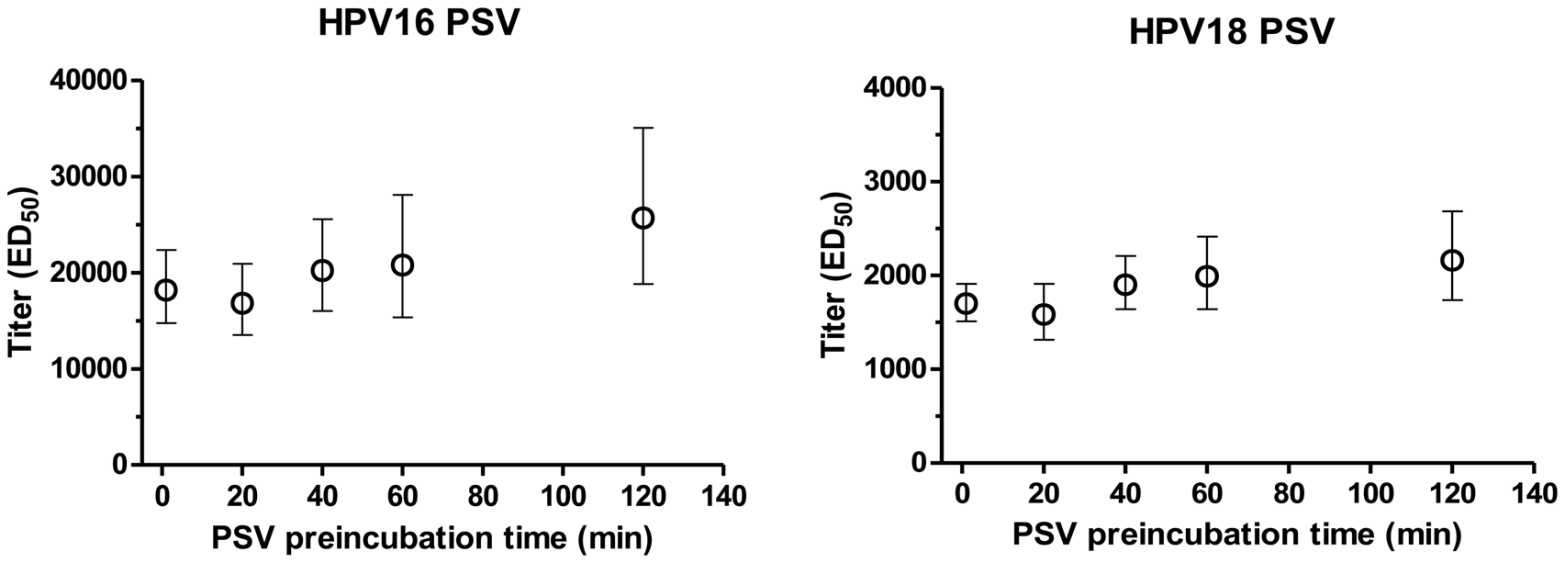
Effect of PSV-serum premix incubation time on neutralization titer. A serum from a Gardasil® vaccinated individual was pre-incubated for different times with HPV 16 or HPV 18 PSVs before the neutralization assay was initiated by the addition of reporter cells. ED_50_ values with 95% confidence intervals are shown.

In the manPBNA described by Pastrana et al., sera and PSV are pre-incubated in a separate plate for 1 hour to ensure complete antibody binding before adding the mix to pre-plated, attached reporter cells. In contrast, for the add-on HT-PBNA trypsin-treated reporter cells are added as a suspension to a mixture of PSV and serum in the cell culture assay plates. To investigate the effect of incubation time of the PSV/serum-premix on the ED_50_-value in the HT-PBNA, our standard serum from a Gardasil® immunized individual was pre-incubated for 2–120 minutes with HPV 16 or HPV 18 pseudovirions before the reporter cells were added. Notably, we found little effect of the PSV-serum pre-incubation time on the ED_50_-value (Figure 5). For all incubation times investigated ED_50_-values did not differ significantly for both HPV 16 and HPV 18 PSVs. This indicates that uptake of the PSV by the added reporter cells is sufficiently slow to allow for antibody binding and neutralization irrespective of incubation time. In the automated HT-PBNA we used a standard pre-incubation time of 1 hour which allows the handling of larger batches of assay plates under identical conditions.

### Effect of High Serum Concentration

At a dilution of 1∶20 we observed that most human sera yielded 60–90% inhibition of bovine papillomavirus 1 (BPV-1) PSV activity in the HT-PBNA, irrespective of whether the sera originated from HPV-vaccinated or non-vaccinated persons. In addition, at a 1∶20 dilution, these sera also neutralized PSV of all other tested HPV types. This effect was not due to reduced viability of the reporter cell line as high serum concentrations rather stimulated cell growth (data not shown). Pastrana and colleagues observed a similar “cross-species inhibition” at 1∶20 dilution for some sera in their SEAP PBNA [16] and consistent with their report, we were also not able to eliminate the inhibition of BPV PSV by heat inactivation, freeze-thaw cycles or centrifugation of the sera. At a dilution of ≥1∶40 none of the sera inhibited BPV PSV activity and sera from vaccinated persons specifically neutralized PSV from vaccine-HPV types or closely related HPV-types only. We therefore conclude that this low titer inhibition is non-specific and not due to cross-neutralizing antibodies.

### Reproducibility of Titers Determined by PBNA

**Figure 6:**
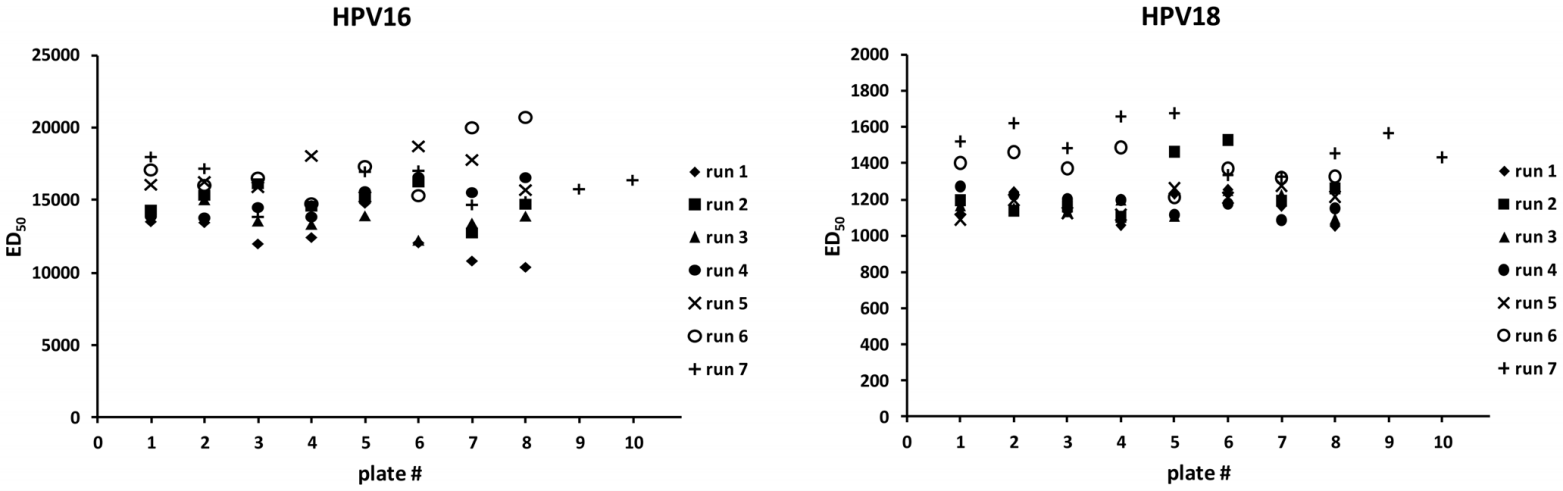
HT-PBNA inter- and intra-run variability of neutralization titers. The ED_50_ values for HPV 16 and HPV 18 PSV of the serum standard were determined in 58 repeats on seven assay days (runs) over a period of 2 months. For six of the 7 assay days, triplicates of the standard serum dilutions were assayed 8 times each, for one assay date the standard serum was assayed 10 times.

The reproducibility of titers obtained with the HT-PBNA for HPV 16 and HPV 18 was determined with our serum standard tested a total of 58 times on individual plates in seven independent runs over a period of two months in the course of a larger serological study (Figure 6). A mean coefficient of variation (CV) of 13% was obtained for the ED_50_-values of 58 repeats with both, the HPV 16 and HPV 18 HT-PBNA. The CV for the intra-run variability with 8–10 repeats per run ranged from 6 to 12%. For a cell-based assay, these CV indicate a low variability. As the underlying assay is newly developed, no data on reproducibility in other laboratories are yet available.

### Assay Application: Detection of Neutralizing Antibodies Derived from Natural Infection

Previously, we analyzed a total of 1271 sera from a different vaccine trial (25% pre-vaccination sera; data not shown) for neutralization of HPV 16, HPV 18 and BPV 1 PSV in the HT-PBNA and found only 8 sera (0.6%) with a BPV 1-specific ED_50_>80 (maximal BPV-specific ED_50_ was 120). Considering the neutralization of BPV 1 in the PBNA as unspecific we defined an ED_50_ of 80 as the cut off value to classify sera positive in the neutralization assay for all HPV types.

**Figure 7:**
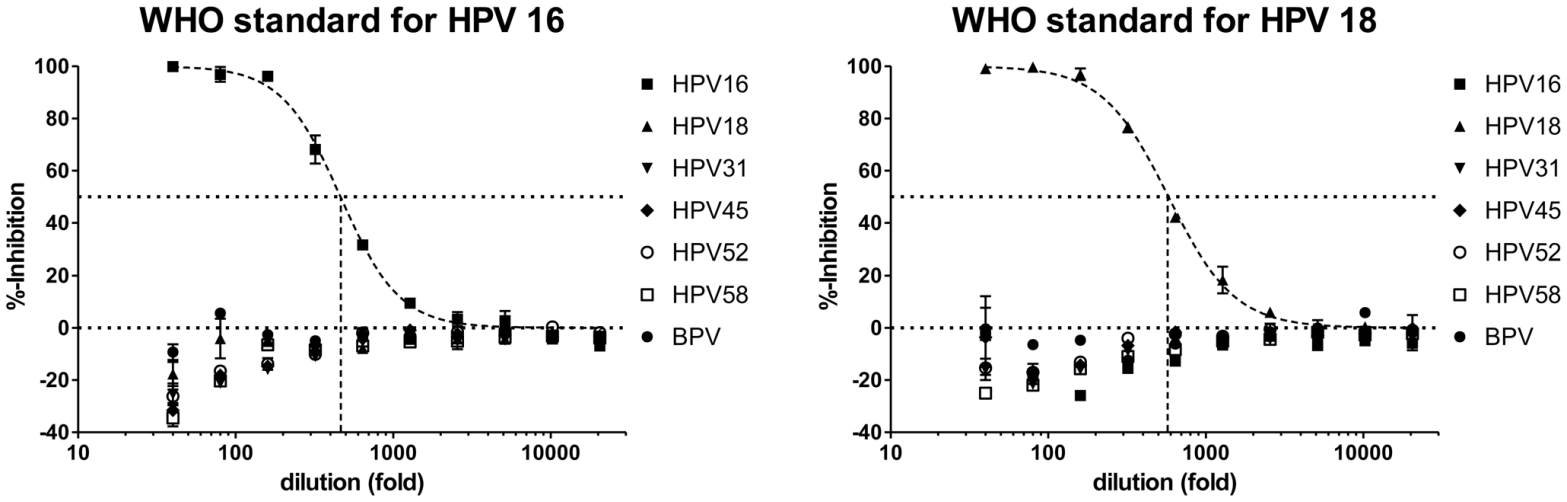
Analytical sensitivity and type-specificity of the HPV 16 and HPV 18 HT-PBNA. Titration of the WHO International Standards for antibodies to HPV 16 (left) and HPV 18 (right) in HT-PBNA using PSV of HPV types 16, 18, 31, 45, 52, 58 and BPV-1.

First, to determine the PV type-specific detection of antibodies in our PBNA we tested the WHO international standards for antibodies to HPV 16 [26] and HPV 18 [27] using PSV of HPV types 16, 18, 31, 45, 52, 58 and BPV 1. The HPV 16 standard neutralized specifically HPV 16 in the HT-PBNA with an ED_50_ of 463 (Figure 7), which is at least five times higher than the titers obtained with the manPBNA in five laboratories participating in the collaborative study to assess the suitability of the serum as an International Standard for antibodies to HPV 16 in immunoassays and pseudovirion neutralization assays [26]. With an assigned potency of 10 mIU/µl for the WHO standard after reconstitution as directed in 0.5 ml distilled water, the neutralization titer of 463 in the HT-PBNA would correspond to an analytical sensitivity of 0.864 mIU. The HPV 18 standard neutralized specifically HPV 18 in the HT-PBNA with an ED50 of 579 (Figure 7) corresponding to an analytical sensitivity of 1.105 mIU.

To compare manPBNA with the HT-PBNA, we tested sera from a German HPV 16 L1-E7 chimeric virus-like particles vaccination trial in CIN patients [17] for neutralization of HPV 16 PSV. In addition, data previously obtained with a Luminex-based total IgG-assay using GST-L1 as an antigen were available for these sera. Thirty-six patients with HPV 16 DNA-positive, high-grade cervical intraepithelial neoplasia (CIN) in groups of 12 participants had received a high- or low-dose vaccine or placebo at weeks 0, 2 and 4. A total of 107 analyzed sera included 35 base line (pre-vaccination) sera and 24 post-vaccination sera each from week 2, 4 and 14 of the study.

**Figure 8:**
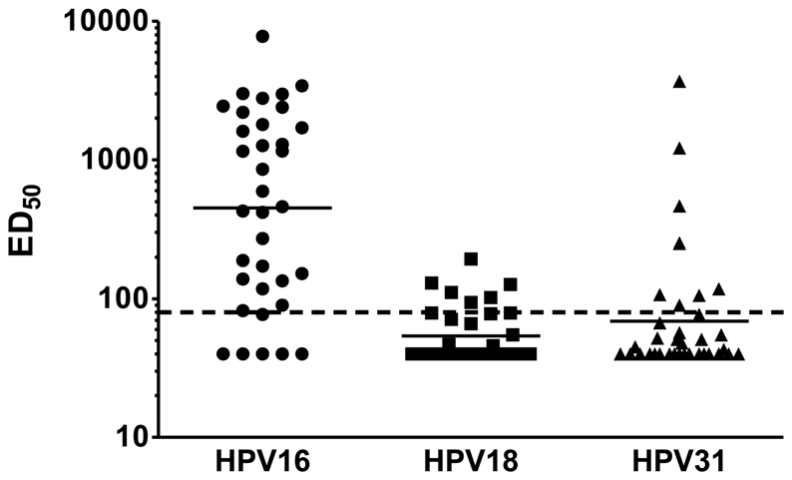
Detection of neutralizing antibodies as result of natural infection by HT-PBNA. Thirty-five pre-vaccination sera from a study involving patients with CIN2+ lesions were tested for neutralizing antibodies against PSVs of HPV types 16, 18 and 31. The geometrical mean titer for each HPV type is indicated as a horizontal line and the cut off value (ED_50_ = 80) as a dashed line.

First, we investigated the performance of the HT-PBNA in detecting neutralizing antibodies as a result of natural infection with 35 available pre-vaccine serum samples using PSVs from HPV types 16, 18, and 31 (Figure 8). All patients from the trial had CIN2+ lesions and were HPV 16 DNA positive at the time of enrollment.

Using an ED_50_ of 80 as cut-off, among the 35 sera tested, 29 (83%), 6 (17%) and 8 (23%) were found positive for neutralization of HPV 16, HPV 18 and HPV 31 PSV, respectively (Figure 8). The geometric mean titer (GMT) of all sera was 449 (GMT of positive sera: 723), 59 (123) and 69 (299) for HPV 16, HPV 18 and HPV 31, respectively. All HPV 18 and HPV 31 neutralizing sera were also positive in the HPV 16 HT-PBNA, with the exception of one serum that was only positive for HPV 18 (ED_50_ = 111). The HPV 31 DNA status of the patients has not been determined, but the well-documented cross-reactivity of HPV 16 antibodies against HPV 31 might explain neutralization of HPV 31. These HPV types are closely related and induction of cross-protection against HPV 31 has been demonstrated by the two commercial HPV-vaccines containing HPV 16 virus-like particles [28]. Interestingly, two sera neutralized HPV 31 PSVs with a 3-fold higher titer compared to HPV 16 PSVs, which highly indicates that the antibodies might be derived from an infection with HPV 31.

**Figure 9:**
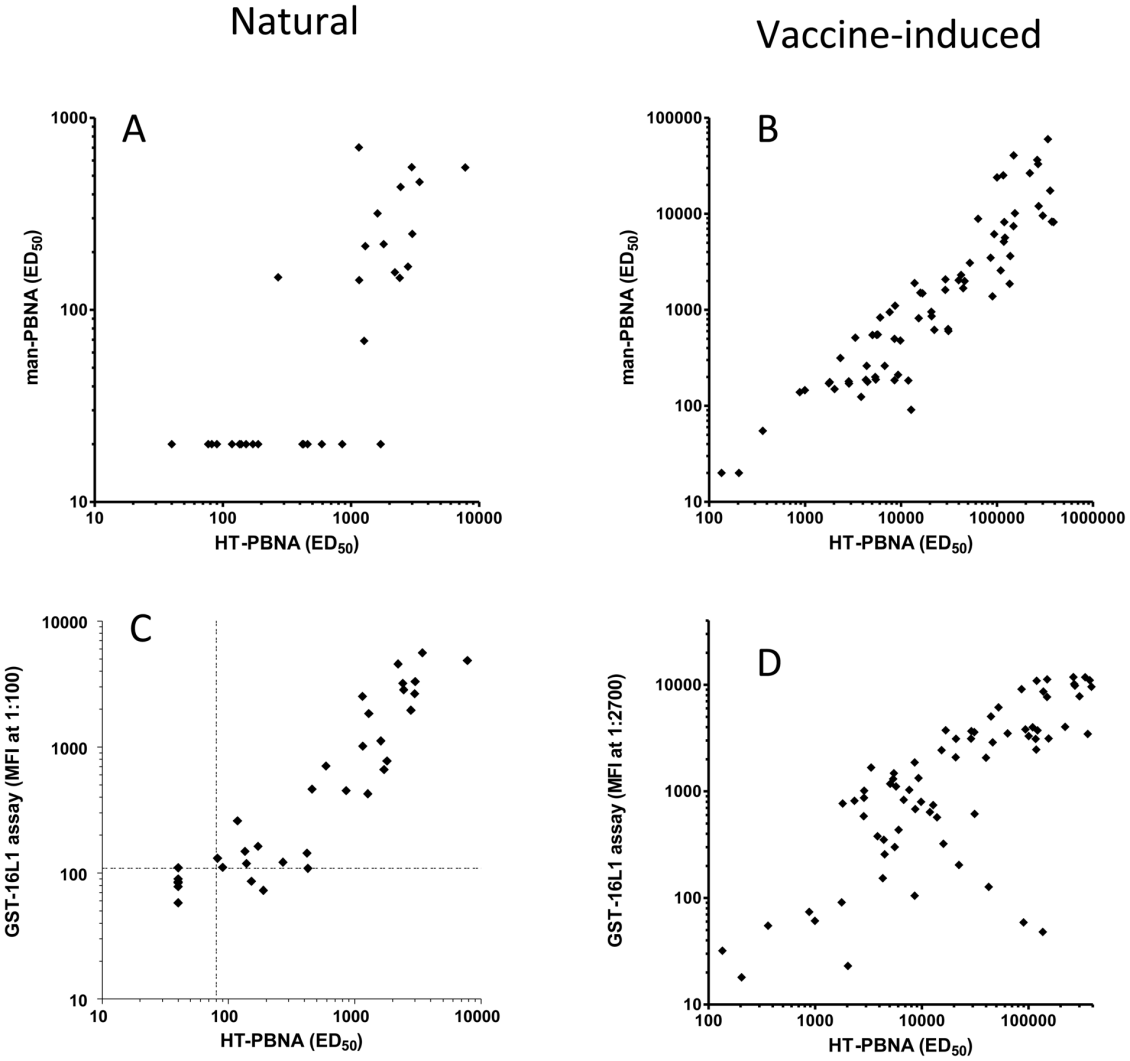
Comparison of HPV 16 HT-PBNA (Gaussia) with manPBNA (SEAP and Gaussia) and GST-HPV 16 L1 antibody binding assay for natural and vaccine-induced HPV16-specific responses. HPV 16 HT-PBNA (A–D), manPBNA using SEAP reporter (A and B; titers, ED_50_) and a bead-based GST-HPV 16 L1 antibody binding assay (C and D; median fluorescent intensity (MFI) at 1∶100 or 1∶2700 serum dilution) were used to determine reactivity of pre- (n = 35; A and C) and post-vaccination (n = 72; B and D) sera. Serum samples analyzed were from women with HPV 16 positive, high-grade cervical intraepithelial neoplasia (base-line sera of the chimeric HPV 16 L1-E7 vaccination trial). Cut off values used for positive/negative classification (broken lines in C) were an ED_50_ of 80 for the HT-PBNA and 109 MFI at 1∶100 for the GST-L1 antibody binding assay.

In general there is a direct, linear correlation between the titers determined in HT-PBNA and manPBNA based on using SEAP, especially for the vaccine-induced high titer sera (Figure 9B). However, the HT-PBNA yields titers about 10-fold higher than the manPBNA (Figures 9A and 9B). In the subgroup of the 35 pre-vaccination sera that contain only antibodies from natural infection, 20 sera were non-neutralizing in the manPBNA. These 20 sera comprise all 6 sera that were negative in the HT-PBNA but also 14 HT-PBNA positive sera with an ED_50_ GMT of 258 and a maximal ED_50_ of 1703. All 15 manPBNA positive sera (GMT (ED_50_) = 250) were also positive in the HT-PBNA (GMT (ED_50_) = 1895). This indicates a higher sensitivity of the HT-PBNA in the detection of sera with low neutralization titers.

The HT-PBNA also showed a good agreement with a Luminex-based total IgG-assay using GST-HPV 16 L1 as antigen (Figure 9C). An R^2^-value of 0.7 (0.67 for the 29 HT-PBNA positive sera only) was determined for the linear regression analysis between GST-HPV 16 L1 antibody reactivity (expressed as median fluorescence intensity, MFI) and the HT-PBNA ED_50_ values. Of the 35 sera, 28 (80%) were considered HPV 16 seropositive by the GST-HPV 16 L1 antibody binding assay (reactivity >109 MFI) and 29 (83%) sera neutralization antibody positive by the HT-PBNA (ED_50_>80). A Cohen’s kappa coefficient of 0.72 indicated good agreement between these two assays.

### Analysis of Post-vaccine Sera

For the 72 analyzed post-vaccination sera of the trial, covering a very wide range of titers, a direct correlation of the manPBNA and the HT-PBNA was observed, but again the HT-PBNA yielded approximately 10 fold higher titers (figure 9B). All except two of the post-vaccination sera were positive in both assays. These two sera were negative in the manPBNA and had low titers in the HT-PBNA with ED_50_-values of 135 and 204, respectively.

As for the low titer pre-vaccine sera, the HT-PBNA and the Luminex-based total IgG-assay showed a good correlation with a R^2^ of 0.61 for the linear regression (Figure 9D). All 72 sera were positive in both assays applying an ED_50_ of 80 as cut off for the HT-PBNA and 10 MFI for the HPV 16 L1 antibody binding assay. Some sera with relative low reactivity in the Luminex assay compared to the HT-PBNA were most probably caused by handling issues in the Luminex experiment as they had high reactivity in both PBNAs.

### Detection of L1 and L2 Vaccine-induced Neutralization Activity Against 7 Papillomavirus Types

**Figure 10:**
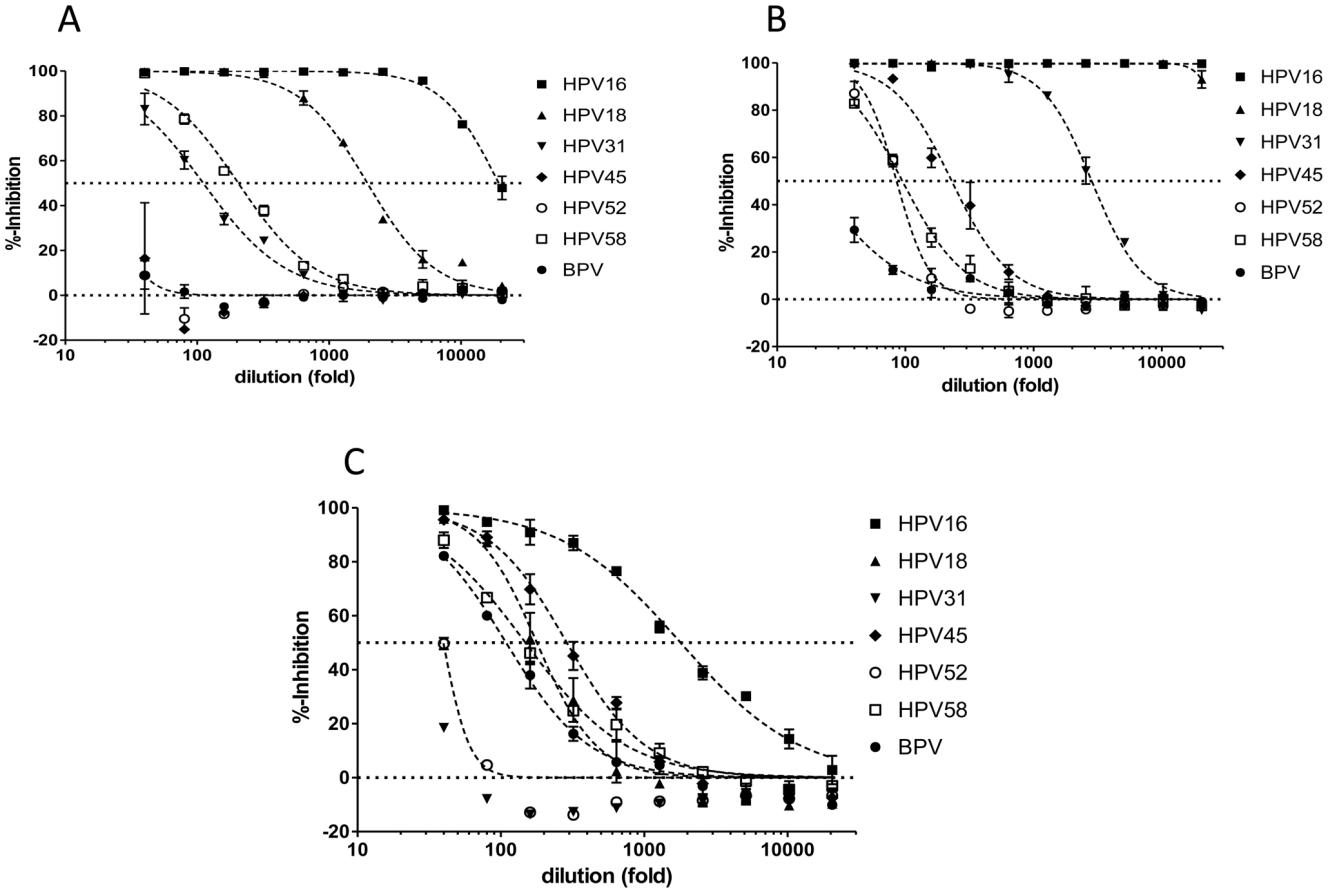
Titration of vaccine sera in the HT-PBNA using PSV of HPV types 16, 18, 31, 45, 52, 58 and BPV-1. A ) Standard serum from Gardasil® vaccinated person. **B**) Cervarix® vaccine serum. **C**) Serum from a mouse immunized with L2 epitopes (amino acids 17–36) from HPV16 inserted in the capsid of adeno-associated virus 2 particles.

An important application of PBNA, ‘the gold standard *in vitro* assay’, for assessing the protective potential of vaccine-induced antibodies, is the detection of cross-neutralization activity against non-vaccine HPV types. The high sensitivity of our HT-PBNA allows the detection of cross-protective antibodies induced by commercial L1-based vaccines as shown in Figure 10A for our in-house standard serum, a collected from a 21 year old female volunteer 3 years after completion of a 3-dose Gardasil® vaccination. The ED_50_ of this serum for HPV 16, 18, 31 and 58 was 19308, 1952, 111 and 205, respectively. No neutralization of HPV types 45 and 52 or BPV-1 PSV was observed.

In Figure 10B the reactivity of a high titer Cervarix® serum in the HT-PBNA is shown. In addition to the vaccine-types 16 and 18 with titers >30,000 also cross-neutralization activity against the non-vaccine HPV types 31, 45, 52 and 58 with ED_50_-values of 2842, 230, 86 and 95, respectively, was detected.

As the PSV are composed of L1 and L2 it is also possible to measure neutralizing antibodies directed against the minor capsid protein L2. In Figure 10C the titration of an experimental serum from a mouse immunized with HPV 16 and HPV 31 L2 (aa 17–36) inserted in the capsid of adeno-associated virus 2 (AAV2) [29] in the HT-PBNA is shown. In addition to the vaccine type HPV 16 PSVs of HPV 18, 45, 58 and BPV are also neutralized by the serum with ED_50_- values of 1796, 179, 289, 149 and 109, respectively. HPV 31 and HPV 58 however, escape this neutralization.

## Discussion

Neutralizing antibodies directed against the L1 major capsid protein play a key role in vaccine-induced immunity against HPV but very likely also in natural immunity. In the past two decades a number of assay systems have been developed and applied to detect L1-specific antibodies. Only some of these assays are able to determine functionality of the antibodies in respect to neutralization of HPV virions. Some of the functional assays include challenge of animals or are so complex that they cannot be applied for routine testing. Because high-throughput assays directly measuring anti-HPV neutralizing antibodies were lacking, surrogate assays such as direct enzyme-linked immunosorbent assays (ELISA) and competitive immunoassays with VLPs as antigen were used [30], [31]. In fact, to date all larger serological-epidemiological and vaccine-monitoring studies have been carried out with non-functional binding assays to monitor anti-L1 responses [32], [33], [34], [35].

There is good reason to believe that anti-L1 antibodies are the key mediators in providing vaccine-induced protection. First, these antibodies have been detected in serum and genital secretion of individuals vaccinated with Gardasil or Cervarix [36]. Next, passive transfer of polyclonal serum and monoclonal antibodies is sufficient to provide protection in mouse and dog challenge models [37], [38]. Finally, the observation that vaccine induced protection is highly HPV type-specific is well in line with the fact that neutralizing antibodies recognize L1 loop structures on the surface of virus capsids and these structures are highly variable among different PV types. In contrast, the remainder of the L1 protein is highly conserved making it very difficult to explain the observed type-specificity if T-cell responses were contributing significantly to protection.

In 2004, Pastrana and colleagues described an *in vitro* pseudovirion-based assay that allowed detection of neutralizing antibodies and this assay is currently considered the gold standard for assaying protective anti-L1 response [16]. It should be noted, however, that to date a formal proof of *in vitro* detection of neutralizing antibodies as a correlate for clinical protection is still missing. The PBNA described by Pastrana et al. quantitatively detects neutralizing antibodies against a number of different HPV types, but the assay is laborious thereby limiting its use in respect to the number of samples, replicates, titration steps and papillomavirus types. To our knowledge the largest study to date has been reported by Dessy et al. [39] in which manPBNA was compared to ELISA and competitive ELISA.

In general, there is a good correlation between non-functional and functional detection of anti-L1 responses when analyzing sera from vaccinees. However, this correlation is the result of (a) the specific induction of conformation-specific antibodies using well-structured antigens with (b) a detection system monitoring antibodies against properly folded antigens. Still, non-functional assays tend to measure a higher degree of cross-type reactivity.

Previously, we demonstrated that a second generation L2 vaccine induces high titers of L2-binding antibodies but only a fraction of these are neutralizing [40]. Detection of functional antibodies will also be required when evaluating cross-protective responses, either against closely related PV types or against naturally occurring variants of the same type.

To fulfill the need for an assay able to detect functional, i.e. neutralizing antibodies, we modified the assay originally described by Pastrana and colleagues. To establish the pseudovirion-based neutralization assayin a high throughput setting, the protocol should be as simple as possible while at the same time sensitivity and reproducibility should be as high as possible. A key parameter for increasing the throughput of our HT-PBNA was the separation of assay plate preparation and performance of the neutralization assay itself. Large batches of 384-well assay plates with serially diluted serum samples can be prepared in advance by automated liquid handling and subsequently stored at −20°C. After thawing, all reagents for the succeeding steps are added consecutively with a bulk dispenser to the assay plates in a pure add-on process, without the requirement for removal of supernatants or pre-incubation on separate plates. With the luciferase from *Gaussia princeps* it was possible to quantify reporter expression with high sensitivity directly in the cell culture plate with high absolute signals and excellent signal to background ratios. Adding the substrate for *Gaussia princeps* luciferase directly to the cell culture plate also avoids the tedious transfer of small volumes of cell culture supernatants to separate luminescence plates, an error-prone step often introducing variation between replicate samples. The compatibility of HT-PBNA with a 384 well format and bulk dispension not only reduces reagent consumption and costs generated by tip dispensing but also accelerates assay assembly and throughput. Furthermore, a high throughput 384 well format is preferable to a manual 96 well format when testing many different pseudovirion types and sera as required, for example, in L2 cross-protection analyses.

Neutralization titers of sera were determined from full titration curves as effective dilutions giving 50% inhibition (ED50). Our standard layout included 10 titration steps, which can be 2-fold, 3-fold or 4-fold. By this, a large range of serum reactivity can be captured, and even high titer immune sera will be fully titrated, a prerequisite for accurate curve fitting. At the same time activity in low titer sera is still determined. To ensure high data quality the curve fit was performed from the median inhibition of triplicates on identical replicates of assay plates. Barcoding of plates enabled complete sample tracking and automated data evaluation with specialized data calculation and management software such as ActivityBase (IDBS).

While we observed a good correlation of HT-PBNA with manPBNA and GST-L1 Luminex assays, the HT-PBNA showed a significant higher sensitivity compared to the manPBNA originally described by Pastrana et al, which uses SEAP as reporter system. Of note, performing the neutralization assay manually but following the protocol for the HT-PBNA we also reached higher sensitivity indicating that the automated processing is not the prime parameter for sensitivity. Still, automation leads to an increase in robustness and decrease in assay variation which itself allows the analysis of low reactive sera. While sensitivity in detecting vaccine-type-specific neutralizing antibodies in vaccinees at early time points after vaccination might not be a critical issue, it becomes very important when analyzing either antibody titers in long-term follow-up studies, natural immunity or vaccine induced cross-protection against non-vaccine HPV types. For example the cross-neutralization induced by Gardasil® and Cervarix® against HPV types 31, 33 and 45 would probably have been missed using the conventional manPBNA. Thus, HT-PBNA delivers more sensitive and therefore comprehensive analyses of natural and vaccine-derived immunity.

As a proof of concept, we demonstrated that HT-PBNA is suitable for detection of L2-directed neutralizing antibodies. As discussed above, functional assays are critical for L2 immunity due to the discrepancy of non-functional and functional assays for this antigen. Recently, Day et al. presented a modified *in vitro* neutralization assay (*L2-assay*) with a much higher sensitivity for L2 but not for L1-specific antibodies [41]. In this assay, pseudovirions are first bound to extracellular matrix on assay plates to induce structural rearrangements in the capsid. Subsequently, L2 is cleaved by addition of furin convertase followed by transduction of cells devoid of heparan-sulfate proteoglycan. These cells can only be infected by pseudovirions that have already been cleaved by furin. In their study, Day and colleagues report 100–200 fold higher L2-specific neutralizing antibody titers compared to the standard assay while sensitivity for L1-specific neutralization is not changed significantly. We did not perform comparison of HT-PBNA and manPBNA sensitivity, although we wanted to confirm that the altered protocol for HT-PBNA is compatible with detection of L2 antibodies. Clearly, even if we assume that HT-PBNA shows also higher sensitivity of detection of L2 antibodies, in the same order of magnitude as we observed for the L1 antibody detection we still are not able to reach the high sensitivity of the L2-assay described by Day et al. or the *in vivo* challenge model described by Roberts et al. [42]. The different incubation steps of this modified L2 assay cannot be readily integrated into the high throughput add-on assay system. Alternatively, Roden and colleagues recently reported that production of pseudovirions in cells overexpressing furin convertase generates pre-cleaved L2 pseudovirions serving as targets for sensitive L2-neutralizing antibody detection (28^th^ International Papillomavirus Conference, Puerto Rico, 2012). These pseudovirions might be very well suited for HT-PBNA, but it remains to be determined whether these modified pseudovirions can be produced for different HPV types.

In conclusion, we present a protocol for a mostly automated high throughput pseudovirion-based assay for the detection of papillomavirus neutralizing antibodies. The increased sensitivity and reproducibility will allow the design of large, comprehensive studies for evaluating natural and vaccine-induced immunity.
